# Leiomyosarcoma of scrotum: case report

**DOI:** 10.11604/pamj.2018.31.238.10741

**Published:** 2018-12-20

**Authors:** Tariq Bouhout, Badr Serji, Ebo Usman Egyir, Benyounes El Amri, Imad Bouhout, Mehdi Soufi, Mohammed Bouziane, Tijani El Harroudi

**Affiliations:** 1 Chirurgie B, CHU Mohammed VI, Oujda, Maroc; 2 Chirurgie A, CHU Mohammed VI, Oujda, Maroc

**Keywords:** Leiomyosarcoma, scrotum, wide excision

## Abstract

Scrotal leiomyosarcoma is rare tumor. It presents as a painless, slow-growing cutaneous lesion. It’s often mistaken for a benign condition. It is best treated by wide local excision. A case of scrotum leiomyosarcoma is presented in a 63 year old patient who was treated for the first time as having a benign lesion.

## Introduction

Leiomyosarcoma of the scrotum is a rare tumor. More than 95% of scrotal sarcomas arise from the spermatic cord, epididymis, or testes, while their location in the scrotal skin is exceptional [1]. It presents as a painless, slow-growing cutaneous lesion. It’s often mistaken for a benign condition with the true diagnosis revealed only on pathologic examination. We report a case of scrotal léiomyosarcome in a 63 year-old man for its rarity.

## Patient and observation

A 63-year-old man with a 3 year history of hypertension, presented with a firm mass in the right hemiscrotum. This lesion had appeared four years previously. On local examination, à 4 centimeters mass was noted on the right scrotum, the left testis, both epididymis and the spermatic cords were normal to palpation, while the inguinal nodes were not palpable.

Mass excision was performed by another surgeon. Pathologic examination revealed a léiomyosarcome of the scrotum, the nodule was well defined and consisted of a proliferation of cells with cigar-shaped nuclei and eosinophilic cytoplasm, the mitotic count was high. The cells stained positive for actin and H-Caldesmon on immunohistochemestry and were negative for desmin. Resected margins were positive.

The patient was referred to our department; he was then evaluated for distant metastatic spread with a total body CT scan that showed no distant metastasis. Wide excision around the scar of the previous excision was performed. Definitive histology showed the absence of tumor on the latter specimen .No recurrence has been recognized 40 months after.

## Discussion

Soft tissue sarcomas are 1% of all malignancies [2]. Leiomyosarcomas constitutes 10 to 20% of soft tissue sarcomas; they arise most often from uterus, gastrointestinal tract and retroperitoneal region [1]. Johnson H Jr in 1987 reported the first known case of leiomyosarcoma of the Scrotum [2]. Only 40 cases have been reported in the literature worldwide till now [3]. Léiomyosarcomes are divided into two types depending on the location, cutaneous léiomyosarcomes arise from the arrectores pilar muscle of the hair follicle or dartos muscle of the genital skin. Subcutaneous léiomyosarcomes arise from the muscle lining of arterioles and veins in the subcutaneous tissue [4]. They present between the fourth and eight decades of life as a painless, slow-growing skin lesion [5]. The duration of symptoms varies from several months to few years. Physical examination exhibits firm, rubbery masses having similar features of a cystic lesion.

This case evinces how a leiomyosarcoma of the scrotum can simulated a benign lesion. He had a lesion that was treated for the first time as being a benign lesion. Resected margins were positive, and that may influence the prognosis of the patient.

Confirmation of the diagnosis of léiomyosarcome is based up on histological examination of biopsy specimen, which reveals spindle cells with cigar-shaped nuclei arranged in interweaving fascicles [1, 6] the diagnosis of malignancy is based on the mitotic rate of 2-10 mitoses/HPF [7], the presence of nuclear pleomorphism, and vascular invasion . On immunohistochemistry, leiomyosarcomas are positive for actine and desmine [1, 3].

It is best treated by wide excision [8], inguinal lymphadenectomy should be performed in those patients with a high degree of suspicion is present for lymph node metastasis. The adjuvant therapy is limited, local control is improved with preoperative or postoperative radiotherapy. The role of chemotherapy is used at several major centers for high-risk patients [3].

The prognosis is generally good in the absence of local recurrence. A positive margin at the first excision increases the risk of local recurrence [4]. Long-term follow-up is needed, because late recurrences and distant metastasis can appear years after the initial excision [3].

## Conclusion

Scrotal leiomyosarcoma is a rare clinical entity; it often resembles a cyst. The recommended treatment of localized leiomyosarcoma of the scrotum is wide excision. Long term follow up is essential, because of the risk of delayed local recurrence and distant metastasis.

## Competing interests

The authors declare no competing interests.
